# Reaching the margins: a study of outreach practices for youth in a NEET-situation

**DOI:** 10.3389/fsoc.2026.1755110

**Published:** 2026-03-26

**Authors:** Ulla-Karin Schön, Joakim Tranquist

**Affiliations:** 1 Department of Social Work, Stockholm University, Stockholm, Sweden; 2 Tranquist Utvardering, Lomma, Sweden

**Keywords:** discretion, NEET youth, outreach work, relational infrastructure, tailored support

## Abstract

This study examines outreach practices within a regional project in Sweden, aimed at supporting young people not in education, employment, or training (NEET). Drawing on Lipsky's theory of street-level bureaucracy, the study examines how frontline staff conceptualize and enact outreach, and how structural conditions shape its implementation. Data was collected through five focus group interviews with 46 practitioners and a document analysis. The findings reveal four interrelated dynamics: (1) outreach as a discretionary practice shaped by fragmented welfare systems, (2) systemic absorption of outreach into ordinary service provision, leading to role drift, (3) absence of relational infrastructure for sustainable transitions, and (4) conceptual vagueness undermining strategic coherence. Although outreach was regarded as essential for engaging socially isolated youth, it remained structurally unsupported and dependent on improvisation and moral commitment. The study extends Lipsky's framework by introducing the concept of relational infrastructure and highlighting the performative dimension of outreach under outcome-driven evaluation regimes. Policy implications include the need for a shared definition of outreach, integrated pathways, and metrics that capture relational outcomes. Outreach, when adequately resourced and strategically embedded, holds transformative potential for promoting social inclusion among marginalized youth.

## Introduction

This study examines the structural conditions shaping outreach work targeting young people aged 15–29 who are not in education, employment, or training (NEET) within Sweden's fragmented welfare landscape. Although the overall proportion of young people in NEET positions has declined from approximately 13.5% in 2006 to 11.2% 2023 across Europe (Eurofound, 2025), the phenomenon has attracted growing attention in both national and international research emphasizing the heterogeneity and complexity of the NEET population (Witte, 2024; Rahmani and Groot, 2023). Young people classified as NEET comprise a diverse group, including both vulnerable and non-vulnerable individuals with varying backgrounds, resources, and life trajectories (Eurofound, 2025). Despite the overall decline in NEET rates, the proportion of young people in NEET positions who experience disabilities, mental health problems or neurodevelopmental disorders, low educational attainment or incomplete schooling, and socio-economic disadvantage has not decreased in recent years (European Commission, 2018). Many of these young people lack access to supportive social networks and may express distrust toward public institutions because of previous negative encounters with welfare systems (Bäckman and Nilsson, 2016; European Commission, 2018). These forms of exclusion are associated with adverse outcomes, including deteriorating health, economic insecurity, and the intergenerational reproduction of disadvantage (Jongbloed and Giret, 2022; Rahmani and Groot, 2023). Moreover, the NEET category has been criticized for individualizing responsibility for exclusion, obscuring structural and institutional barriers to participation and downplaying broader societal responsibility (McPherson, 2021; Wrigley, 2024).

Recent research indicates that the Swedish welfare state has undergone intensified restructuring, producing institutional conditions that significantly shape the discretionary space within which outreach work is conducted (Andersson, 2013; Brauer, 2025). Studies show that youth policies increasingly construct young people in NEET positions as vulnerable subjects within a welfare system characterized by declining coherence and limited capacity to deliver accessible and integrated support (Jonsson et al., 2022). Concurrently, long-term transformations have reduced welfare universalism, increased social and territorial inequalities, and shifted responsibility for service provision downward to the municipal level. This has resulted in substantial variation in local resources, organizational capacity, and institutional responses (Brauer, 2025). These developments intersect with the consolidation of activation-oriented labor market policies (Jonsson et al., 2022). Activation is a dominant and expanding policy paradigm across OECD countries, sustained through ongoing recalibration of disciplinary and enabling mechanisms (Brauer, 2025). In Sweden, activation reforms have increasingly emphasized conditionality and individual responsibility, while governance arrangements have become more fragmented and subject to frequent reorganization (Andersen and Larsen, 2024). Together, these institutional changes shape both the expectations placed on outreach initiatives and the working conditions of frontline practitioners.

However, previous research highlights persistent shortcomings in the ability of these existing support measures to reach the most vulnerable individuals in NEET positions (Jongbloed and Giret, 2022; Rahmani and Groot, 2023). Many young people in this situation are not registered with public welfare institutions and do not seek assistance on their own initiative (Eurofound, 2025). This institutional “invisibility” underscores the need for proactive forms of intervention. Outreach work, defined as the active identification and engagement of young people within their own social and spatial contexts, is increasingly recognized as a crucial strategy for reaching those in the most marginalized NEET positions (European Commission, 2018). While outreach is frequently promoted as a key approach for engaging marginalized youth, existing research indicates that its implementation has been uneven and often lacks a clear theoretical foundation, particularly in relation to vulnerable young people in NEET positions (Smoter, 2022; Nsour et al., 2020).

Effective outreach strategies are grounded in the practice of direct engagement, where outreach workers immerse themselves in the lives of individuals and communities (Hureau and Papachristos, 2025). Outreach work has gained attention as a proactive and relational strategy for engaging individuals on their own terms (McPherson, 2021). This involves visiting the areas where young people in NEET positions live, and collaborating with local institutions that are already working with these populations. Outreach aim to build trust between service providers and the target population, while also supporting individuals at risk of social exclusion in overcoming barriers such as stigma or past negative experiences of services (Nsour et al., 2020). Research illustrates that outreach strategies must be responsive to the needs and life situations of the intended recipients to be effective (Andersson, 2013). Dewson et al. (2006) describe four types of outreaches, each reflecting a different approach to engagement. “Detached” outreach occurs in informal settings where potential target populations tend to gather, such as streets, gallerias, clubs, or railway stations. “Domiciliary” outreach takes place in the homes of individuals, providing support in familiar and private environments. “Peripatetic” outreach involves outreach workers cooperating with community-based institutions, including schools, community centers, and local non-governmental organizations. Finally, “satellite” outreach is organized through information centers located in geographically remote or underserved areas (Dewson et al., 2006). Beyond the mode of delivery, Smoter (2022) distinguish between “preventive” and “remedial” outreach strategies. Preventive strategies are typically employed at early stages, often during the time of education/school presence, and aim to address risk factors before they lead to marginalization and social isolation. These proactive interventions are described as more cost-effective and impactful than efforts to re-engage individuals who have already become marginalized. In contrast, remedial strategies focus on those who are already facing significant challenges, seeking to reconnect with services and support. Evidence further emphasizes the importance of early intervention (Svedberg et al., 2020; Atkinson, 2010). The longer a young person remains outside education or employment, the more difficult re-entry becomes, and the higher the risk of long-term social exclusion, deteriorating mental health, and reduced life chances (OECD, 2016; Högberg et al., 2020). Proactive, outreach work is described as offering a promising avenue for re-engagement, given that it is adequately resourced, locally anchored, and integrated within a broader, coordinated welfare strategy (Svedberg et al., 2020; Atkinson, 2010). Central to successful outreach is also the establishment of trust-based relationships that relies on relational continuity, empathy, and the ability of professionals to work without rigid bureaucratic constraints. Previous research illustrates that outreach programs are most effective when they are flexible, individualized, and integrated across sectors, offering holistic support that extends beyond mere labor market activation (Hall et al., 2015; Atkinson, 2010). In this context, cross-sectoral collaboration between schools, municipalities, health services, and civil society actors becomes essential for young people in a NEET position.

Young people in NEET positions often remain invisible to welfare institutions due to fragmented services, weak coordination, and increasing activation-oriented policy demands. Although outreach is frequently highlighted as a key strategy for engaging marginalized youth, little is known about how outreach workers interpret their mandate, navigate organizational constraints, and exercise discretion within these shifting welfare structures. Recent transformations of the Swedish welfare state marked by decentralization, conditionality, and performance pressures further complicate the practical conditions for proactive engagement. This creates a gap between policy expectations and the everyday realities of outreach work. Research has examined youth marginalization and inter-agency collaboration, but the situated practices of outreach workers remain underexplored. This study addresses these gaps by analyzing how frontline staff conceptualize and enact outreach, and how structural conditions shape their possibilities for supporting young people who are disconnected from welfare services.

### Aim

The aim of this study is to examine how outreach work is conceptualized and carried out by frontline staff in Sweden, and to identify the structural conditions that shape their practice and influence service delivery to marginalized individuals in NEET positions. Using the theory of street-level bureaucracy, the study analyzes how outreach workers navigate discretion, resource constraints, and organizational expectations in their everyday work.

### Theoretical foundation

Lipsky's theory of street-level bureaucracy provides a foundational lens for analyzing outreach work with young people in NEET positions. In Lipsky's formulation, public policy is ultimately shaped not only by legislation or organizational mandates but by the discretionary judgments and coping practices of frontline workers operating under conditions of uncertainty, resource constraints, and ambiguous objectives (Lipsky, 1980). Although outreach workers are not always formally situated within bureaucratic hierarchies, they are nevertheless embedded in institutional mandates, funding arrangements, and accountability structures that define their scope of action. As Evans and Harris (2004) argue, discretion extends beyond formal decision-making authority and includes the interpretive and relational work required to navigate complex welfare environments. This positions outreach workers as hybrid actors who are simultaneously constrained by bureaucratic expectations and required to engage in relational, trust-based practices at the margins of organizational systems. In the context of youth policy, street-level bureaucracy has been widely applied to examine practices that unfold in semi-formal or liminal institutional spaces where policy aims, organizational structures, and young people's lived realities intersect. Outreach workers supporting young people in NEET situations may operate at such intersections. Identifying, engaging, and building trust with young people who may avoid or distrust institutional contact require substantial interpretive flexibility and responsiveness. At the same time, outreach workers must navigate performance pressures, shifting policy priorities and fragmented welfare infrastructures. These conditions may deepen the discretionary space described by Lipsky and amplify the tensions between bureaucratic expectations and the relational, adaptive work required in outreach.

Recent developments in street-level bureaucracy research further support this expanded interpretation. Scholars have highlighted that discretion is not solely an individual cognitive process but is enacted relationally, emotionally, and spatially (Dubois, 2010; Zacka, 2017). Discretion unfolds through interactions characterized by affect, recognition and moral judgment, and is shaped by the temporal rhythms of relationship-building. Such insights may be particularly salient for outreach workers, who must cultivate trust and continuity with young people who are often socially isolated or have experienced failure interacting with welfare services. Complementing this perspective, relational social work and spatial theory deepen our understanding of outreach as a practice embedded in social relations and physical settings. Andersson (2013) conceptualizes outreach as a contact-creating and resource-mediating practice enacted through iterative phases: initiating contact, building trust, supporting motivation, and linking individuals to services. This process relies on relationality, temporal pacing, and the outreach workers' sensitivity to young people's circumstances. Spatial theory further highlights that outreach is shaped by the environments in which it unfolds. Public, semi-public, and private spaces offer different possibilities and constraints for engagement: public spaces may facilitate accessibility but limit confidentiality; private homes require greater relational capital and procedural care (St Croix and Doherty, 2023).

## Materials and methods

This study adopts explorative qualitative methods. The focus is to understand how outreach is enacted, experienced, and made meaningful by those involved in its delivery. Given the localized and context-specific nature of outreach, a qualitative approach enables an in depth understanding of the perspectives of the frontline practitioners and their managers.

The context of the study is a European Social Fund project coordinated across seven regional Coordination Associations in Stockholm, bringing together the Public Employment Service, the Social Insurance Agency, Health Services, and 20 municipalities. The project targets individuals with complex needs and weak educational or labor-market attachment, offering tailored support through approaches such as Supported Employment, Supported Education, and Case Management. One core component of the project is outreach work aimed at identifying and engaging individuals not reached by traditional welfare services. The project seeks to improve access to coordinated support for young people categorized as NEET by strengthened outreach efforts. Project staff were employed within one of the participating welfare organizations, either working full-time in the project or splitting their time with regular duties. Most were social workers, case managers, or work coaches.

### Data collection

Data collection through focus group discussions and document analysis allows for the triangulation of empirical insights. Focus groups provide a space for staff to reflect collectively on their experiences, revealing shared understandings, challenges, and strategies in practice, while document analysis offers complementary information on formal project ambitions, operational guidelines, and reporting mechanisms. Purposive sampling ensured that all staff directly involved in outreach were represented, thereby strengthening the relevance and richness of the data. The empirical material consists of five focus group discussions with six–eleven participants. A total of 46 staff on different levels of the project participated in the FGI's all with outreach work as part of their job responsibilities. Additionally, an analysis of project-related documents such as the original project application to ESF, internal guidelines and progress reports, was conducted. The focus groups took place between September 2024 to April 2025, either in person or via digital platforms. All sessions were recorded and transcribed verbatim. The interviews focused on topics such as definitions of outreach, experiences of outreach work, perceived barriers and enablers, role expectations, inter-agency collaboration, and reflections on impact. Project documentation provided complementary insights into formal ambitions and operational practices.

### Data analysis

Thematic analysis was employed as the primary analytical method, following the six-step guide proposed by Braun and Clarke (2021): (1) familiarization with the data, (2) generating initial codes, (3) searching for themes, (4) reviewing themes, (5) defining and naming themes, and (6) producing the report. Coding was conducted iteratively by the two authors who initially worked independently and later synthesized their insights. Emerging themes were organized into categories reflecting different aspects of outreach practice: the conceptual framing of outreach, organizational conditions, relational dynamics, coping strategies, and implications for service outcomes. NVivo software was used to assist in the coding process (2–4).

### Ethical considerations

The study followed the ethical standards outlined by the Swedish Science Council. All participants received written and verbal information about the study's purpose, their rights, and the voluntary nature of participation. Written or recorded oral consent was obtained from all participants. Data were anonymized during transcription, and identifiers were removed from the final report. The researchers maintained a reflexive stance throughout the process, acknowledging their prior role as follow-up researchers in the project and the relational dynamics this entailed. The study was approved by the Swedish Ethical Review Authority Dnr: 2024-02826.

## Results

The analysis of the focus group interviews reveals how outreach is currently practiced, as well as the underlying challenges, tensions, and potential for development. Four central themes emerged from the material: (1) *The discretionary nature of outreach work*, (2) *Systemic absorption of outreach into ordinary service provision*, (3) *Lack of institutional infrastructure for sustainable transitions*, and (4) *Conceptual vagueness of outreach as a strategy*. The themes will be presented in more detail below.

### The discretionary nature of outreach work

The participants consistently described outreach as both “necessary” and “underdeveloped”. Outreach was understood as essential for reaching young people who were “invisible to the welfare system”, often living in social isolation and experiencing mental ill-health, neurodevelopmental conditions, and an unfinished upper-secondary education. At the same time, outreach was described as difficult to operationalize to these young people, because it involves engaging individuals whose situations are uncertain, fluid, and not fully legible to institutional categories.

Definitions of the intended outreach target group varied considerably among staff, depending on local context, professional background, and organizational positioning. Some participants emphasized those completely outside the welfare systems, while others included individuals “circulating intermittently” between activities and programs. This variability reflects what Lipsky (1980) describes as the expansion of discretion under conditions of ambiguous policy goals and limited guidance. Staff are required to translate abstract ambitions, such as “reaching the invisible”, into situated judgments about whom to prioritize, when to intervene, and how persistently to engage.

Despite outreach being a stated ambition of the project, its implementation was described as “highly uneven”. Some staff had developed proactive efforts, collaborating with schools, NGOs, or community actors, while others relied primarily on referrals from social services. This often resulted in a reactive rather than proactive model, in which individuals had to demonstrate some degree of motivation or institutional visibility in order to access support. As one participant explained:

“*Most of the people we work with, or at least some of them, aren't really NEETs in full sense. They're partially NEET, but they're involved in some kind of activity. They might spend some time at [activity], then move to [the municipality], and then we take over. After that, they go to [activity], spend a year there, and then come back. And these individuals are, to some extent, resourceful—they're able to ask for help. They move between different programs. And then we have the 'real' NEETs, the ones who are just sitting at home. Those are the ones we really need to reach somehow.”*

This distinction illustrates how discretion is exercised through categorization practices that are not formally codified but emerge in everyday work (Dubois, 2010). Prioritizing “reachable” individuals can be understood as a coping strategy in Lipsky's sense, allowing workers to manage uncertainty and workload while still maintaining a sense of professional efficacy. At the same time, this practice risks reproducing exclusion by systematically sidelining those whose needs are the most complex.

### Systemic absorption of outreach into ordinary service provision

The prioritization of “reachable” individuals was also shaped by organizational pressures and increasing demand for services. The participants emphasized that although outreach was initially central to their assignment, it was gradually displaced by the growing number of incoming referrals and administrative responsibilities. As the project became more integrated into ordinary service provision, outreach work was increasingly crowded out by case management, job coaching, and bureaucratic tasks. One of the outreach workers expressed;

“*We do everything. /…/ But when you don't have the time, and we have an incredible number of participants each… Then that's [outreach work] what you start cutting back on. Because your focus is on the participants, and you do everything for them. We're supposed to find employers and do everything else too. /…/ So, the outreach work ends up getting less and less. There was more of it in the beginning [of the project].”* (FGI1)

This process of absorption reflects a structural paradox: outreach is promoted in policy discourse as proactive and innovative yet undermined in practice by organizational logics that prioritize outputs, documentation, and measurable outcomes. The dual roles described by many staff, being both outreach workers and job coaches, were particularly difficult to reconcile. Outreach was described to require temporal flexibility and relational continuity, whereas employment-oriented work was governed by performance indicators and time-limited interventions.

As waiting times and eligibility barriers in other parts of the welfare system increased, staff described how they increasingly “carried” participants instead of linking them onward:

“*We are supposed to link people to services, but because of waiting times there, we have to continue supporting them ourselves.”* (FGI3)

This illustrates how outreach is transformed from a bridging practice into a compensatory one, filling gaps left by fragmented welfare provision. In Lipsky's (1980) terms, this role drift reflects how street-level workers adapt to structural constraints by redefining the scope of their work. Rather than being a failure of outreach, this absorption can be understood as a rational and ethically motivated response to institutional scarcity, albeit one that undermines the long-term sustainability of outreach as a distinct practice.

### Lack of institutional infrastructure for sustainable transitions

Closely related to the previous theme, participants repeatedly emphasized the lack of organizational structures that could support transitions from outreach into long-term support. While informal collaboration between individual professionals sometimes enabled successful handovers, these arrangements were described as fragile, person-dependent, and difficult to sustain.

The outreach workers described situations where substantial relational work such as building trust, motivating participation, and re-establishing contact, was followed by organizational breakdown when attempting to connect individuals to other services. Motivation could quickly dissipate if not met with timely and coordinated responses. As one participant noted:

“*You could spend a lot of time on outreach, but if there's no one on the receiving end, then it doesn't matter….we make no real difference.”* (FGI1)

This finding highlights the importance of what we conceptualize as relational infrastructure: the institutionalized conditions that allow relational work to translate into durable support. Drawing on Andersson (2013) understanding of outreach as a contact-creating and resource-mediating process, relational infrastructure refers to shared principles, routinized handover procedures, and inter-organizational trust that enable continuity across phases of engagement. Without such infrastructure, outreach risks becoming what participants described as a “box-ticking exercise”, disconnected from meaningful outcomes. Importantly, relational infrastructure does not eliminate uncertainty but seem to help professionals manage it. By providing predictable pathways and timely responses, it reduces the risk that relational investments made under uncertain conditions collapse at points of transition.

### Conceptual vagueness of outreach as a strategy

The final theme that emerged from the analysis concerns the conceptual ambiguity surrounding outreach within the project. Although outreach was frequently referenced in policy documents and internal discussions, participants described it as an “unfinished idea”, lacking shared definitions, methodological guidance, and clearly articulated objectives.

“*We may not have clearly conveyed how our outreach approach is connected to the idea of inclusion.”* (FGI5)

Participants expressed a desire for clearer strategic frameworks, training, and shared learning processes. However, their accounts also suggest that what was sought was not rigid standardization, but institutional recognition of outreach as a legitimate, relational, and time-intensive practice. The difficulty of aligning outreach with outcome-based performance indicators, focused on employment or education transitions, further contributed to its marginalization. Relationship-building, trust, and stabilization were experienced as central outcomes, yet remained largely invisible in formal evaluations. This conceptual vagueness mirrors what Zacka (2017) describes as the moral and interpretive labor of street-level workers operating in normative gray zones. Outreach workers are required to act without fully specified criteria for success, relying instead on professional judgment and relational knowledge. Attempts to fully operationalize outreach may therefore conflict with its underlying logic, which depends on openness to contingency and responsiveness to individual life situations.

## Discussion

This study examines how outreach is conceptualized and enacted by frontline practitioners within a regional ESF project targeting young people who are not in education, employment, or training (NEET). Across the five focus groups, staff portrayed outreach as both indispensable and as structurally precarious, highly contingent on individual discretion, improvised workarounds, and moral commitment rather than durable organizational support. The findings illuminate a persistent tension between policy rhetoric and the realities of implementation. Building on Lipsky's (1980) theory of street-level bureaucracy, the analysis shows how outreach, celebrated in policy discourse, risks becoming performative under outcome-driven evaluation regimes, unless supported by clear strategy, adequate resources, and cross-sectoral coordination, which goes in line with previous studies (Brodkin, 2011; van Berkel et al., 2017; Andersen and Larsen, 2024).

The findings illustrate how policy is made in practice through the discretionary judgments and coping repertoires of frontline workers who operate amid ambiguous mandates and resource constraints (Lipsky, 1980). The practitioners reported prioritizing “reachable” or “motivated” young people and routinizing activities that were more legible to managerial oversight. This mirrors what Evans and Harris (2004) identify as the persistent and necessary elasticity of discretion in social work, and what Dubois (2010) and Zacka (2017) conceptualize as the moral and affective labor, embedded in front-line encounters. In contexts of high caseloads, limited time, and diffuse accountability, selectivity may become a rational response. Such practices may inadvertently reproduce inequalities by sidelining those with the most complex needs. In effect, outreach workers enact policy-by-proxy by translating vague ambitions, such as “reach the invisible”, into workable practices that fit local constraints, shaping inclusion as well as exclusion at the point of delivery (Brodkin, 2011; van Berkel et al., 2017).

The results illustrate how outreach, intended as a bridge into existing services, is absorbed into ordinary provision and gradually transforms into a substitute for overstretched or inaccessible systems. Staff described shifting from short, relationally intensive linking to long-term, compensatory support, “carrying” participants due to waiting lists, fragmentation, or categorical eligibility barriers. This role drift resonates with analyses of activation governance in which universalist ideals are recalibrated under managerialism and conditionality, pushing street-level organizations to buffer mismatches between policy promises and available provision (Brodkin, 2011; Andersen and Larsen, 2024). In Sweden, ongoing rescaling, decentralization and layering have further widened territorial and organizational disparities, intensifying the local burden of orchestration (Brauer, 2025). The result is a paradox: outreach is valorized as a proactive strategy, yet its practical conditions erode as staff are pulled into case-management, job-coaching and compliance tasks that are easier to count and attribute to performance indicators.

To make sense of why successful initial engagement often failed to translate into sustained inclusion in welfare services, we introduce relational infrastructure: the shared principles, routines, referral protocols and trust relations across organizations that enable young people to move seamlessly from contact, to assessment, to appropriate support. Previous studies show that effective outreach depends on continuity, pacing and trust (Andersson, 2013). But these features require both relational skills at the micro level and the meso-level connective tissue across schools, municipalities, social and health services, and civil society (Hall et al., 2015; European Commission, 2018; Saltkjel et al., 2021). The places where outreach occurs offer different possibilities for confidentiality, dignity, and follow-up. Domiciliary engagement is often pivotal for reaching highly isolated youth, but it presupposes robust safeguards and coordinated pathways on the “receiving end” (Dewson et al., 2006; St Croix and Doherty, 2023). Where relational infrastructure is thin, outreach becomes precarious “window-dressing”: it raises expectations and briefly mobilizes motivation without being able to guarantee timely access to suitable, coordinated services. The present findings thus complement and extend Lipsky by specifying the organizational conditions under which discretion fosters inclusion rather than drift: discretion must be scaffolded by relational infrastructure to be developmentally meaningful.

The participants described outreach as an “unfinished idea,” lacking common definitions, methodological guidance, and measurable objectives. This ambiguity matters because outcome-driven regimes tend to privilege measurable results, thereby systematically undervaluing forms of work that are slow, relational, and processual (Brodkin, 2011). When performance indicators privilege entries to employment/education over intermediate relational milestones, workers are incented to reallocate time from proactive search and trust-building to reportable tasks. The result is symbolic compliance, doing what “counts”, rather than what counts for the most excluded youth (McPherson, 2021; van Berkel et al., 2017). The results of this study illuminate this displacement. Conceptual clarification is therefore not merely an academic nicety; it is a precondition for aligning metrics, mandates, and methods in ways that legitimate and protect outreach's distinctive contribution in practice (European Commission, 2018; Hall et al., 2015). The results from this study verify and nuance several strands of prior work. In relation to modes and timing of outreach, staff emphasized the centrality of domiciliary outreach for “hidden” NEETs, consistent with Dewson et al.'s (2006) typology and with evidence that home-based engagement reduces barriers for youth with low trust or mobility. The results also confirm previous studies that emphasize the need to balance preventive school-proximate outreach with remedial efforts for those already detached (Atkinson, 2010; Smoter, 2022; Svedberg et al., 2020). The longer the disconnection, the steeper the re-entry gradient and the more likely co-morbid mental health challenges, underscoring why proactive, persistent outreach pays off only when embedded in coordinated pathways (OECD, 2016; Högberg et al., 2020; Svedberg et al., 2020). Effective outreach programs are flexible, relational and inter-organizational, offering holistic support beyond labor market activation (Hall et al., 2015; European Commission, 2018; Saltkjel et al., 2021). The findings in this study show how such cross-sectorality is aspirational without formalized handovers, shared assessment frames, and guaranteed follow-up slots.

The drift toward activation and conditionality reported in this study is consistent with research on activation regimes across Europe, where conditionality and performance pressures shape front-line triage and target-group selection (van Berkel et al., 2017; Andersen and Larsen, 2024). In the Swedish context, recent rescaling and fragmentation magnify local variation and put a premium on street-level improvisation (Brauer, 2025; Jonsson et al., 2022).

The study contributes new knowledge in two ways; first, by showing how evaluation metrics shape discretionary routines, the study elaborates a performative dimension of street-level work: what is counted (and countable) steers who is reached and how (Brodkin, 2011). Second, the proposed concept of relational infrastructure specifies the meso-institutional conditions under which discretion can support developmental engagement rather than short-term manageability. In other words, the problem is not discretion *per se*, but discretion without a connective architecture that guarantees continuity, reciprocity and timeliness across organizational boundaries (Andersson, 2013; Saltkjel et al., 2021; European Commission, 2018). Taken together, these insights extend Lipsky's framework by clarifying how governance instruments (metrics, mandates) and organizational ecologies co-produce front-line practice in outreach to NEET youth.

### Implications for policy and practice

Translating these insights into practical implications reveals several priority areas. In line with other studies, a priority is to develop a shared operational definition of outreach, one that is tied to concrete methods and clear criteria for identifying target groups, as this reduces the tendency for efforts to drift toward already “reachable” participants (Dewson et al., 2006; European Commission, 2018; Andersson, 2013). A second priority concerns the importance of relational infrastructures: cross-agency handover routines, shared assessments, warm referrals, no-wrong-door access, and timely follow-up mechanisms appear to support continuity and help sustain motivation once contact has been established (Hall et al., 2015; Saltkjel et al., 2021). Systems oriented primarily toward measurable transitions may undervalue relational work; several studies therefore point to the usefulness of complementary indicators, such as trust, continuity of contact, stabilization, service attachment, and access to health services and to the need to ensure that outreach time is not crowded out by more easily reportable tasks (Brodkin, 2011; van Berkel et al., 2017). Another recurring finding is that combining outreach with case management or job-coaching roles can lead to role drift; separating or sequencing these functions, supported by manageable caseloads and clear handover points, is described as one way to mitigate this risk (Andersen and Larsen, 2024; Hall et al., 2015). Finally, research on “hidden” NEETs underscores the relevance of domiciliary and place-based outreach, highlighting the need for safe and ethical home-based practices (Dewson et al., 2006; St Croix and Doherty, 2023).

### Methodological reflections and limitations

As a qualitative study centered on staff experiences within one regional ESF project, transferability is necessarily bounded by institutional context. The absence of young people's own voices is a limitation, given that perceived legitimacy and trust are co-constructed in interaction. The absence of young people's voices clearly limits the insight into how outreach is experienced by its intended beneficiaries and how effective it is from their standpoint. In addition, the timing, a year into the project, may understate later maturation or learning effects. Future research should (i) incorporate youth perspectives, (ii) adopt longitudinal designs to track transitions beyond initial engagement.

### Conclusion

The study advances the understanding of why outreach, despite its intuitive and political appeal, so often under-delivers for the most marginalized youth. Outreach becomes transformative when the micro-level craft of relational work is scaffolded by meso-level relational infrastructure and macro-level metrics that recognize slow, developmental change. The contribution to theory is twofold: specifying how outcome regimes render outreach and proposing relational infrastructure as the connective architecture that allows discretion to foster inclusion rather than merely manage scarcity.

## Data Availability

The datasets presented in this article are not readily available because Informed consent only includes availability to researchers. Requests to access the datasets should be directed to the corresponding author(s).
